# Health commodities logistics management information system performance at public health facilities of Amhara region, Ethiopia

**DOI:** 10.3389/fpubh.2025.1545429

**Published:** 2025-03-24

**Authors:** Zelalem Tilahun Mekonen, Denny J. Cho, Teferi Gedif Fenta

**Affiliations:** ^1^Department of Social and Administrative Pharmacy, School of Pharmacy, College of Health Sciences, Addis Ababa University, Addis Ababa, Ethiopia; ^2^Logistics Department, Kyrgyz State Technical University, Bishkek, Kyrgyzstan

**Keywords:** LMIS, evaluation, performance, health commodities, health facilities

## Abstract

**Introduction:**

Ensuring equitable access to essential medicines is a major global health challenge, particularly in low- and middle-income countries. Effective supply chain management and Logistics Management Information Systems (LMIS) are crucial for addressing these challenges. Despite substantial efforts, significant LMIS implementation issues continue, especially in Ethiopia.

**Objectives:**

The study aimed to evaluate the performance of health commodities logistics management information systems at public health facilities in the Amhara Region, Ethiopia.

**Methods:**

The study conducted in public health facilities of the Amhara region in Ethiopia used a quantitative methods approach. The region is supplied by four Ethiopian Pharmaceutical Supply Service hubs. A total of 102 facilities, selected through stratified random sampling, were included in the study. Data were collected through record review and and observation using data abstraction checklists to evaluate the LMIS forms availability, utilization, supply, and report timeliness.

**Results:**

Infrastructure challenges were noted, including inconsistent power supply and limited internet access, with only 42.2% having internet connectivity. LMIS performance varied, with high availability and utilization of forms like the IFRR and RRF but lower rates for some forms and digital systems. Reporting and feedback mechanisms were generally adequate, though only 37.3% of facilities received periodic written feedback from higher levels of the healthcare system.

**Conclusion and recommendation:**

The evaluation of the LMIS in Amhara Region’s public health facilities shows notable achievements in the widespread use of LMIS forms and reporting systems. However, ongoing challenges such as unreliable infrastructure, poor internet connectivity, and insufficient human resources impede effective LMIS performance. Addressing these issues, digitalization of the LMIS, strengthening feedback mechanisms, and supervisory support will enhance LMIS performance and improve health outcomes.

## 1 Introduction

Equitable access to health commodities, particularly essential medicines remains a critical global health challenge, particularly in low- and middle-income countries, where access issues persist despite the growing burden of diseases (1, 2). Affordable health commodities reduce mortality and morbidity (%^3^–^5^). Governments must ensure access to health commodities including essential medicines, vaccines, and supplies as part of the right to health, which is essential for achieving Sustainable Development Goal 3 (%^6^–^9^). Despite these objectives, a significant portion of the global population needs consistent access to essential health commodities, leading to substantial financial burdens on healthcare systems in developing countries (4, 10, 11).

Effective supply chain management is pivotal in global health systems to ensure the availability and accessibility of essential health commodities (2). In low-income countries, the supply chain for health commodities faces challenges rooted in structural and health system complexities, contributing to inefficiencies. These include multiple tiers of stock management and decision-making processes that exacerbate complexities and inefficiencies (12, 13). Robust logistics management information systems (LMIS) are essential for effective supply chain management, ensuring the availability of medicines, vaccines, and health technologies (%^14^–^16^). The LMIS is an essential tool that enables the real-time flow of logistics information, providing vital support for strategic decision-making at every level of healthcare (5, 17). Challenges such as information distortions and lack of integration can lead to significant disruptions in the supply chain, known as the bullwhip effect (18, 19).

Programs like the Integrated Pharmaceutical Logistics System (IPLS) and digital LMIS initiatives have demonstrated potential impact in enhancing the effectiveness of LMIS in countries such as Ethiopia, although significant implementation challenges persist (20, 21).

Despite numerous efforts, various studies have indicated that the current health commodities’ LMIS implemented in Ethiopia’s healthcare system faces several challenges. These include partial implementation and a lack of sufficient LMIS forms in health facilities (Shewarega (22, 23)), poor infrastructure such as internet, electric power, and computers (24), challenges with LMIS interoperability with other systems (25), poor data quality (20, 26, 27), lack of end-to-end data visibility (14), gaps in training and staff commitment (28) and inadequate supportive supervision and timely feedback from higher levels (29).

Addressing these challenges necessitates comprehensively evaluating the current LMIS implementation (14, 30). The objective of this study is to conduct a comprehensive performance evaluation of the current health commodities LMIS implementation in public health facilities across the Amhara Region of Ethiopia.

## 2 Methods

### 2.1 Study area

**Figure 1:**
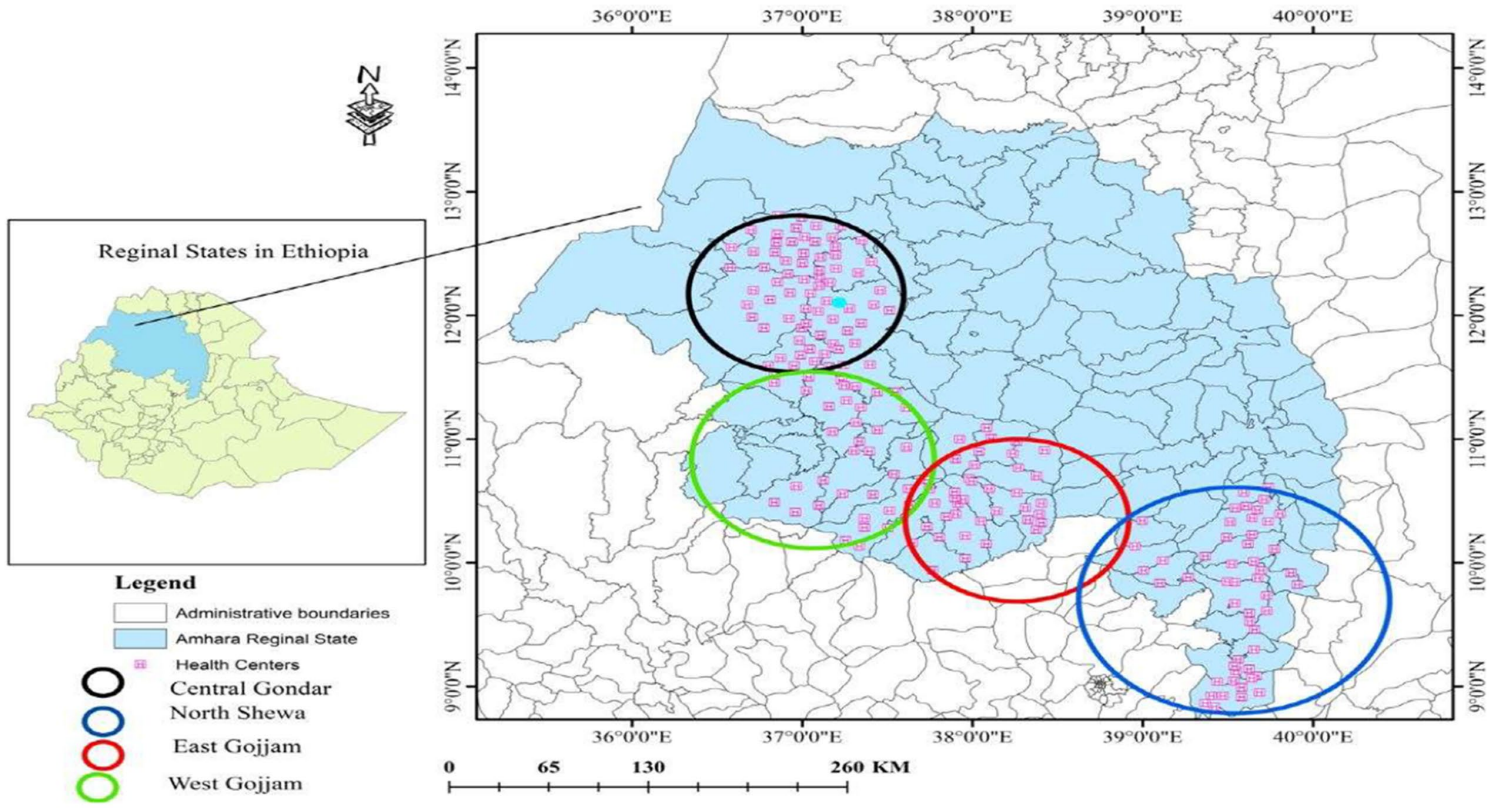
Map of selected health facilities for the study in Amhara Region, March 2022.

The study was conducted in the Amhara National Regional State of Ethiopia, the second-most populous region in the country. It is administratively divided into 12 zones and three city administrations. As of 2021, the region had 5,775 functional health facilities, including 87 public hospitals, 873 public health centers, 3,565 health posts, six private hospitals, and 1,244 private clinics. Public health facilities in the region are primarily supplied through four hubs of the Ethiopian Pharmaceutical Supply Service (EPSS; Figure 1).

### 2.2 Study design

The study employed an institution-based quantitative design. This research is part of a larger project focused on assessing the performance of the health commodities LMIS, with its results mainly based on quantitative data analysis.

### 2.3 Source and study population

The study included all public health facilities managing health commodities for the provision of healthcare service. Specifically, the study population comprised selected public health facilities in the region.

### 2.4 Sampling procedure

Health centers and hospitals in all zonal administrations, except those with security problems, were eligible for sampling. Six zonal and two city administrations were chosen and grouped into three clusters based on EPSS hub locations. Ultimately, four zones and two city administrations were included. As per USAID’s Logistics Indicators Assessment Tool (LIAT) recommendation, 15% of facilities were sampled, with a final adjusted sample size of 102. The sample size was allocated proportionally across Zones and *Woreda* (district level administration), using a multi-stage stratified random sampling method based on the number of facilities.

### 2.5 Inclusion and exclusion criteria

#### 2.5.1 Inclusion criteria

- All Zonal Administrations
- All public health facilities that have been functional for more than 1 year

#### 2.5.2 Exclusion criteria

- Health facilities damaged in conflict-affected areas or had security problems during the study.
- Health Posts

### 2.6 Data collection process

Data was collected through record review, after requesting the responsible persons to locate the appropriate document in each department and observations conducted by experienced and well-trained pharmacists using data abstraction checklists. These checklists were adapted from the Logistics Indicator Assessment Tool (LIAT), Logistics System Assessment Tool (LSAT), and the Ministry of Health’s supply chain monitoring and evaluation tools (31, 32).

### 2.7 Data quality assurance

Ten experts in the field face-validated the data collection tools. Additionally, data collectors participated in a two-day training session. The tools were pre-tested, and adjustments were made based on the feedback received.

### 2.8 Operational definitions

- **RRF (Report and Requisition Form)**: This LMIS tool allows health centers and hospitals to report consumption and request resupply of health commodities.
- **IFRR (Internal Facility Reporting and Requisition Form)**: This tool facilitates the requisition and redistribution of health commodities within health facilities used by health centers and hospitals.
- **HPMMR (Health Post Monthly Report and Resupply Form)**: This LMIS tool allows health posts to report consumption and request resupply of health commodities from health centers.
- **Model 19/Health**: An official financial transaction tool used in health facilities for receiving health commodities from suppliers, with a serial registration.
- **Model 22/Health**: An official financial transaction tool used in health facilities for issuing health commodities to other facilities and units, with a serial registration.
- **Health Commodities Management Information System (HCMIS)**: It is a digital LMIS used to manage and track health commodities across the public healthcare system in Ethiopia.

### 2.9 Data analysis and interpretation

The data were reviewed for completeness and internal consistency before being entered into Epi Info Version 7 for initial processing. The data were then exported to SPSS Version 23.0 for detailed data management and analysis.

## 3 Results

### 3.1 General information

**Figure 2:**
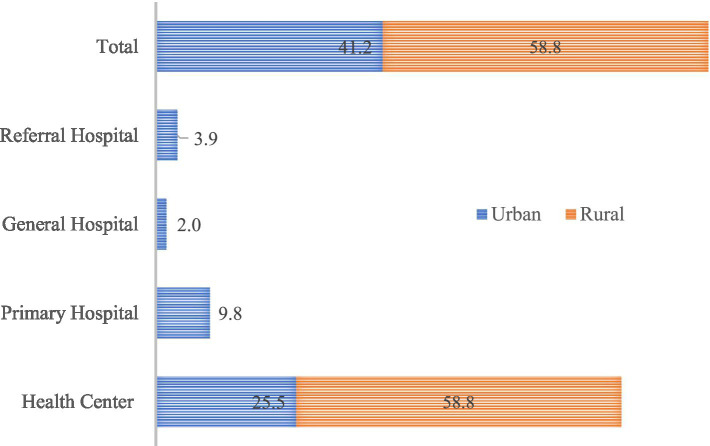
Percentage distribution of surveyed health facilities by level and location, March 2022.

The study surveyed 102 public health facilities, of which 58.8% were located in rural areas. The majority of these facilities were health centers, 84.3% followed by primary hospitals 9.8% (Figure 2).

**Figure 3:**
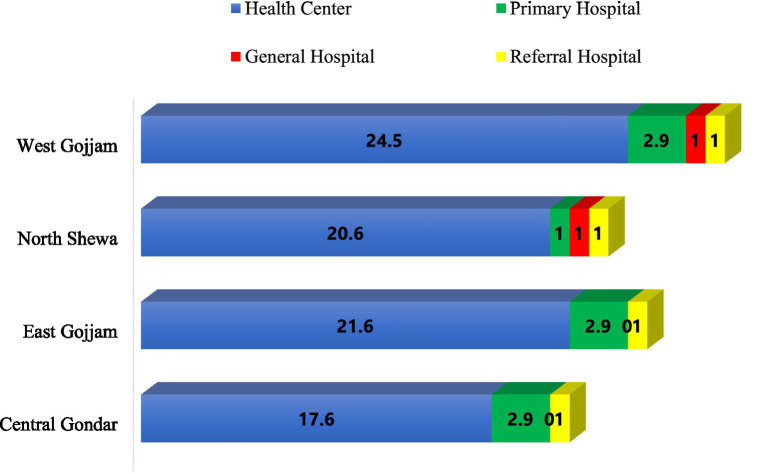
Percentage zonal distribution of surveyed public health facilities, March 2022.

The health facilities were distributed across four zonal administrations: West Gojjam (29.4%), East Gojjam (25.5%), North Shewa (23.5%), and Central Gondar (21.6%; Figure 3).

The study involved 19 rural Woredas (woreda is the third-level administrative hierarchy in Ethiopia and a primary unit for local governance, equivalent to a district in other countries) and 9 City and Town administrations. On average, the distance of surveyed health facilities from the Woreda and zonal town was 11.98 km ± 9.56 and 63.13 km ± 60.7, respectively. The average distance from the regional city and the supplier EPSS hub was 149.58 km ± 146 km and 96.6 km ± 82.5 km, respectively. Additionally, the mean service duration of the health facilities was 19.4 years ±16.9 years, and each facility was connected to an average of 4.9 ± 2.07 lower-level facilities through the referral system (Table 1).

**Table 1:** Geographic location, year of service, and number of referral facilities for surveyed health facilities in Amhara Region, March 2022.

SN	Variable	Median	Mean ± SD	Min	Max
1.	Distance of HF from Woreda Town (KM)	9	11.98 ± 9.56	0	38
2.	Distance of HF from Zonal Town (KM)	42	63.13 ± 60.7	0	222
3.	Distance from Regional City (KM)	122.5	149.58 ± 146	4	690
4.	Distance from the supplier EPSS Hub (KM)	70	96.6 ± 82.5	1	265
5.	Service duration (years)	12	19.4 ± 16.9	1	85
6.	Number of referral health facilities	5	4.9 ± 2.07	1	11

### 3.2 LMIS-related infrastructure

Among the surveyed health facilities, 98 (96.1%) had an electric power supply on the day of the visit, but 28 (28.6%) experienced power interruptions during the study visit. Backup generators were available in 63 (61.8%) of the facilities. Internet connectivity was accessible in 43 facilities (42.2%), with only 25 (58.1%) having access specifically in the pharmacy department. The types of internet connectivity reported included wireless (55.8%), broadband (44.2%), and data (4.7%).

Half of the health facilities needed dedicated computers for the health supply chain and LMIS activities in key areas like dispensaries, stores, supply chain coordinator offices, and ART pharmacies. The other 50% had at least one computer for these activities, with a median of 0.5, a mean of 1.1 ± 2.1, and a range from 0 to 13 (95% CI [0.68, 1.5]). For the manual LMIS system, only 63 (61.8%) of the facilities reported having allocated budgets for printing and duplicating LMIS formats.

### 3.3 Performance of supply chain-related initiatives

Health facilities had an average of 7.05 ± 6.07 dispensaries (95% CI [5.86, 8.24]). The average number of pharmacy personnel present during the visit was 5.36 ± 8, ranging from 1 to 56 per facility. Only 62 (60.8%) facilities had a designated health commodities supply chain management coordinator. On average, 1.65 ± 1.34 professionals (95% CI [1.38, 1.91]) were assigned to supply chain activities, ranging from zero to eight. The average number of finance professionals assigned to supply chain tasks was 0.95 ± 2.44 (95% CI [0.43, 1.48]).

Of the healthcare facilities surveyed, 83 (81.4%) received program and RDF products directly from EPSS hubs within their respective clusters, while the remaining facilities received their supplies through the Woreda Health offices. Specifically, 56 (54.9%) facilities were supplied by the Bahir Dar EPSS hub, 24 (23.5%) by the Addis Ababa EPSS hub, and 22 (21.6%) by the Gondar EPSS hub.

**Figure 4:**
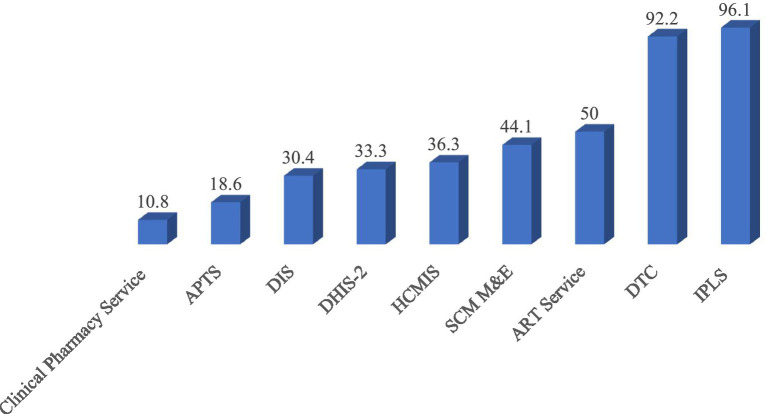
Implementation status of pharmacy services and supply chain management initiatives at public health facilities in the Amhara Region, March 2022.

During the visit, the three most widely practiced pharmacy service and supply chain management initiatives were the Integrated Pharmaceutical Logistics System (APTS), Drug and Therapeutics Committee (DTC), and ART Service, which were operational in 98 (96.1%), 94 (92.1%), and 51 (50%) of the facilities, respectively. In contrast, the least implemented initiatives were the Drug Information Service (DIS), APTS, and clinical pharmacy service, with frequencies of 31 (30.4%), 19 (18.6%), and 11 (10.8%), respectively (Figure 4).

### 3.4 Availability, utilization, and supply of LMIS forms

There is a notable variation in the availability and utilization of LMIS forms among healthcare facilities. The IFRR and RRF demonstrated the highest availability and utilization, were present in 99 and 98% of facilities, respectively, and were almost fully utilized in nearly all facilities. In contrast, the HCMIS Guide/Manual had the lowest availability, present in only 18.6% of health facilities, and was utilized in 84.2% of those cases. The Bin Card (BC) was widely available in 96.1% of health facilities, of which 89.8% actively used it, and 87.8% maintained a supply for three or more months. Although the Stock Record Card (SRC) was less available, present in only 27.5% of health facilities, it had a high utilization rate of 92.9% in the facilities where it was available. Other forms, such as the Job Aids for IPLS (laminated), Quantification Tool (Updated), and Physical Inventory form, also exhibited high utilization rates (Table 2).

**Table 2:** Availability, utilization, and supply of LMIS forms in healthcare facilities in Amhara, March 2022.

SN	LMIS forms in HFs [*N* = 102]	Availability [*N* = 102]		Utilization status		Supply for 3+ months		Supply for < 3 months	
		N	%	N	%	N	%	N	%
1.	Bin Card (BC)	98	96.1	88	89.8	86	87.8	12	12.2
2.	Stock Record Card (SRC)	28	27.5	17	60.7	26	92.9	2	7.1
3.	HPMMR in HCs [*N* = 86]	72	83.7	65	90.3	63	87.5	9	12.5
4.	IFRR	101	99	100	99	86	85.2	14	14.9
5.	RRF	100	98	100	100	90	90	10	10
6.	Model 19/H	47	46.1	43	91.5	44	93.6	3	6.4
7.	Model 22/H	49	48	44	89.8	46	93.9	3	6.1
8.	IPLS SOP	63	61.8	57	90.5				
9.	Physical Inventory form	80	78.4	75	93.8				
10.	Job Aids for IPLS (laminated)	38	37.3	37	97.4				
11.	HCMIS Guide/Manual	19	18.6	16	84.2				
12.	Quantification tool (Updated)	63	61.8	61	96.8				
13.	M&E tool	54	52.9	48	88.9				

In the evaluation of LMIS forms at 19 APTS sites, it was found that Cash Sales Tickets, Daily Summary Sheets, and Sales Ticket Pad Registers were universally available at all sites, achieving 100% availability. The Free Registration Book, however, had the lowest availability, being present at only 89.5% of the sites. Utilization across these forms was notably high, with Cash Sales Tickets, Daily Summary Sheets, and Sales Ticket Pad Registers all being used at 100% of the sites. Most APTS-related LMIS forms had sufficient supplies of printed copies that lasted three or more months (Table 3).

**Table 3:** Availability, utilization, and supply of LMIS forms in APTS sites in Amhara, March 2022.

SN	LMIS forms in APTS Sites [*N* = 19]	Availability		Utilization status		Supply for 3+ months		Supply for < 3 months	
		N	%	N	%	N	%	N	%
1.	Cash sales Ticket	19	100	19	100	18	94.7	1	5.3
2.	Free registration book	17	89.5	17	100	17	100	–	–
3.	Credit registration book	18	94.7	18	100	18	100	–	–
4.	Daily summary sheet	19	100	19	100	18	94.7	1	5.3
5.	Monthly sales summary sheet	18	94.7	18	100	18	100	–	–
6.	Model 19/H	18	94.7	17	94.4	18	100	–	–
7.	Model 22/H	18	94.7	17	94.4	18	100	–	–
8.	Sales ticket pad register	19	100	19	100	18	94.7	1	5.3
9.	Cash delivery note	18	94.7	18	100	18	100	–	–

In the assessment of LMIS forms at 51 ART sites, the ART monthly activity report format and the ART register were highlighted for their exceptional availability at 98% of the sites. In contrast, the EDT form had the lowest availability, found at only 17.6% of the sites. The utilization status of LMIS forms was greater than 80% across all ART sites. Furthermore, the ART register and the patient information sheet (PIS) had a better supply of print copies for managing supplies lasting three or more months at the ART sites (Table 4). Challenges related to the availability and utilization of LMIS forms directly impact LMIS performance, as these forms are critical for record-keeping, transactions, and reporting. A shortage of LMIS forms and gaps in their utilization lead to poor data quality and management, which in turn directly affects LMIS performance and subsequently impacts supply chain efficiency. Quality data and effective performance in LMIS are essential for informed decision-making. Therefore, gaps in this area directly impact inventory policies, order quantities, inventory costs, and ordering costs, ultimately affecting the availability of health commodities and service delivery.

**Table 4:** Availability, utilization, and supply of LMIS forms in ART sites in Amhara, March 2022.

SN	LMIS forms in ART Sites [*N* = 51]	Availability		Utilization status		Supply for 3+ months		Supply for <3 months	
		N	%	N	%	N	%	N	%
1.	ART Monthly Activity report format	50	98.0	44	88.0	44	88.0	6	12.0
2.	EDIT	9	17.6	8	88.9	8	88.9	1	11.1
3.	Patient tracking chart	38	74.5	31	81.6	34	89.5	4	10.5
4.	ART Register	50	98.0	45	90.0	46	92.0	4	8.0
5.	Patient Information Sheet (PIS)	44	86.3	39	88.6	42	95.5	2	4.5

### 3.5 Health commodities LMIS performance

Among all surveyed facilities, only 37 (36.3%) had adopted the digital LMIS system known as the Health Commodities Management Information System (HCMIS), a facility version named Dagu. Of these, 21 (56.8%) utilized Dagu version 2.0, while 16 (43.2%) used the older version, Dagu 1.0. The average duration of HCMIS implementation preceding the visit was 4.63 ± 3.38 years (95% CI [3.5, 5.75]). Interestingly, the majority (73%) were in the mature implementation phases, with the remaining 27% evenly split between the pre-intensive and intensive phases.

**Figure 5:**
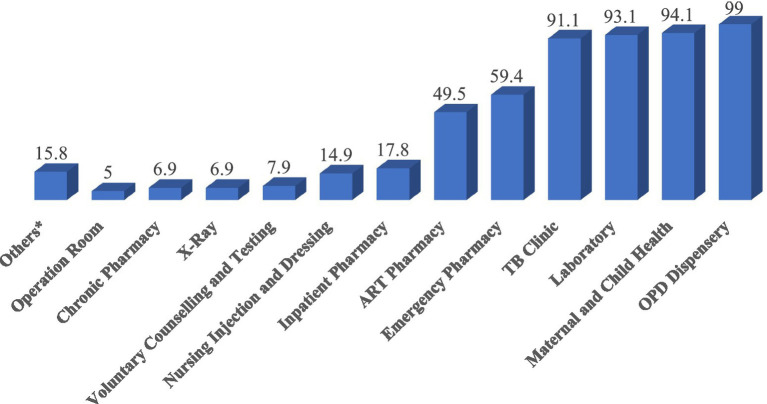
Utilization status of IFRR in public health facilities of Amhara Region, March 2022.

Nearly all health facilities, except one, were found to utilize IFRR during the visit. The outpatient pharmacy, Maternal and Child Health pharmacy, and laboratory department were the primary units using IFRR to report consumption and request resupply from the health facility store, with frequencies of 100 (99%), 95 (94.1%), and 92 (93.1%), respectively. In contrast, the X-ray unit, Chronic diseases management pharmacy, and Operation room were the least frequent users of IFRR, with frequencies of 7 (6.9%), 7 (6.9%), and 5 (5%), respectively (Figure 5).

Among facilities utilizing IFRR, 96 (95%) adhered to a two-week schedule for reporting consumption and requesting resupply from the store. In contrast, 2 (2%) operated on a facility-specific one-month schedule, and 3 (3%) did not have a specified schedule. Among these facilities, 60 (61.2%) displayed their IFRR schedule at the store, while the remaining 38 (38.8%) did not.

Of the 719 dispensary units expected to report according to the IPLS 2-week schedule, 482 units (67.04%) submitted their latest IFRR on time. The median number of dispensaries submitting on time was 5, with a mean of 4.92 ± 3.3 and a range from 0 to 20 (95% CI [4.3, 5.6]). Conversely, 26.8% of dispensary units submitted their IFRR after the scheduled date, with a median of 1, a mean of 2.14 ± 5.8, and a range from 0 to 53 (95% CI [0.93, 3.4]).

Among the 719 dispensaries, 518 (72.1%) had their supply requests fulfilled within 2 days of making the request. Store managers mentioned workload, the complexity of requests needing additional processing time, and delays in IFRR reporting from units as factors contributing to delays in meeting the two-day supply request timeframe.

Of the 406 health posts connected via the referral system to the 86 surveyed health centers, 283 (69.7%) submitted the Health Post Monthly Report and Resupply Form (HPMRR) to the health centers during the latest reporting period. The median number of health posts submitting the HPMRR on time was 3, with a mean of 3.29 ± 1.91 and a range from 0 to 8 (95% CI [2.9, 3.7]). Among these, 202 (71.4%) health posts submitted the HPMRR on schedule by the fifth day after the reporting period (Table 5).

**Table 5:** Reporting status of IFRR and HPMRR at public health facilities in Amhara Region, March 2022.

SN	Variable	Frequency n(%)	Mean ± SD (Min, Max)	95% CI
1.	DUs sent IFRR as per the schedule (*N* = 719)	482(67.04%)	4.92 ± 3.3 (0,20)	[4.3,5.6]
2.	DUs sent IFRR after the schedule (*N* = 719)	193(26.8%)	2.14 ± 5.8(0,53)	[0.93,3.4]
3.	Stores resupply to DUs within 2 days of request? (*N* = 719)	518(72.1%)	5.34 ± 6.4(0,59)	[4.1,6.63]
4.	Health Posts submitted the latest HPMRR to the HC (*N* = 406)	283(69.7%)	3.29 ± 1.91(0,8)	[2.9, 3.7]
5.	Health Posts submitted the latest HPMRR as per the schedule (*N* = 283)	202 (71.4%)	2.69 ± 1.85(0,6)	[2.3,3.1]

Out of 102 health facilities, 100 (98%) used the Report and Requisition Form (RRF) for bi-monthly reporting of consumption and resupply requests to EPSS and Woreda Health Offices (WoHO). The remaining two facilities did not use the RRF due to difficulties in understanding its use and workload constraints. Among the RRF reports reviewed over the year, only 450 (75%) were approved and signed by either the health facility heads, Chief Executive Officers, or Chief Clinical Officers. The number of approvals per facility had a mean of 4.5 ± 2.2, ranging from 0 to 6 across the six reports reviewed during the year (95% CI [4.06, 4.94]).

**Figure 6:**
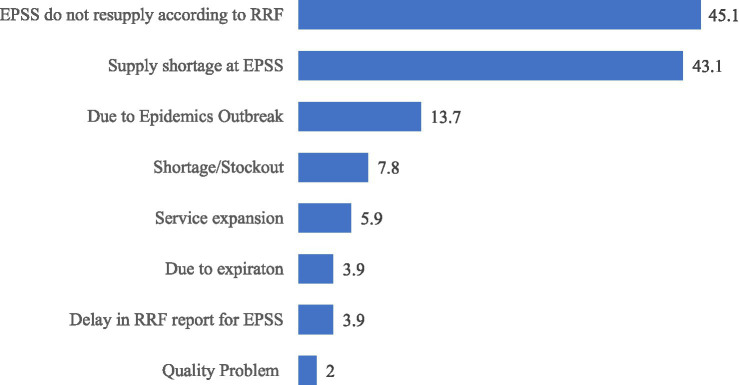
Reasons for emergency order placement in public health facilities of Amhara Region, March 2022.

Among the 100 facilities using the RRF, 51(51%) placed emergency orders at least once during the year. These orders had a mean of 2.32 ± 7.5, ranging from 0 to 72 (95% CI [0.83, 3.8]). The main reasons for these emergency orders were issues related to EPSS, including shortages and difficulties in fulfilling the requested quantities as specified in the RRF (Figure 6).

### 3.6 LMIS reporting and feedback

**Figure 7:**
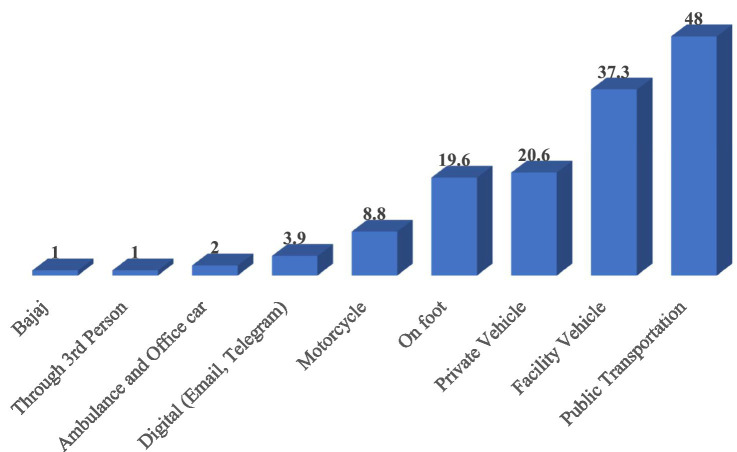
Frequently used transportation methods for sending LMIS reports to higher levels in public health facilities of Amhara Region, March 2022.

Regarding LMIS report submission channels, 77 health facilities (75.5%) submitted their reports to the Woreda Health Office (WoHO). Additionally, 62 facilities (60.8%) sent reports to EPSS, 16 facilities (15.7%) to the Zonal Health Department, and three facilities (2.9%) to the Regional Health Bureau. The most frequently mentioned transportation methods for sending LMIS reports to higher levels were public transportation, used by 49 facilities (48%); facility vehicles, used by 38 facilities (37.3%); and private vehicles, used by 21 facilities (20.6%; Figure 7).

**Figure 8:**
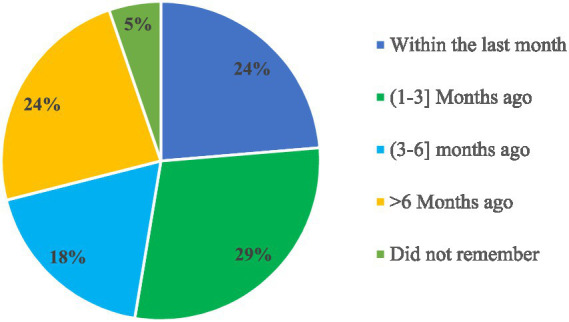
Timeliness of written feedback on LMIS reports in public health facilities of Amhara Region, March 2022.

Among the facilities surveyed, 38 (37.3%) received written periodic feedback on supply chain management and LMIS reports from higher levels. This feedback came from various sources, including 29 facilities (76.3%) received it from the Woreda Health Office (WoHO), four facilities (10.5%) from Zonal Health Departments, two facilities (5.3%) from partners, and three facilities (7.9%) from integrated teams. Of those receiving feedback, nine facilities (23.7%) received it within the month before the visit, while 11 facilities (28.9%) received it between 1 and 3 months prior to the visit (Figure 8).

### 3.7 LMIS supportive supervision

**Figure 9:**
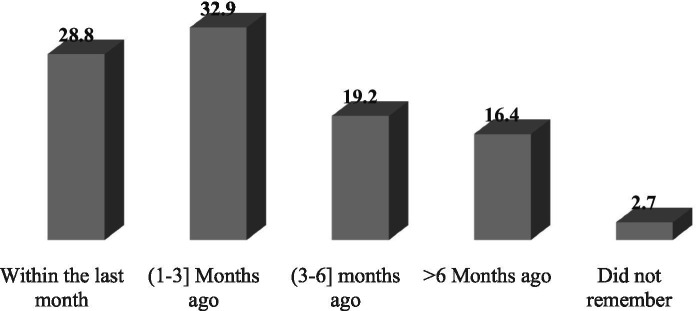
Supportive supervision of public health facilities on LMIS in Amhara Region, March 2022.

In the year leading up to the study, only 73 out of the surveyed health facilities (71.6%) received supportive supervision for supply chain activities and LMIS-related issues. Of these, 21 facilities (28.8%) received supportive supervision within the month prior to the visit, while 24 facilities (32.9%) received it between 1 and 3 months before the visit (Figure 9).

Recent supportive supervision was provided by the following institutions, including Woreda Health Office (WoHO) in 38 facilities (52.1%), Zonal Health Departments in 23 facilities (31.5%), Regional Health Bureaus in 15 facilities (20.5%), partners in 11 facilities (15.1%), and integrated teams in 4 facilities (5.5%). This supervision was reported to have improved supply chain management and LMIS practices, focusing on enhancing APTS, DAGU, DIS, DTC, and IFRR reporting. Additionally, it claimed to result in better bin card management, more effective RRF reporting systems, improved data quantification, and better management of expired products and stockouts.

## 4 Discussion

The evaluation of the Logistics Management Information System (LMIS) in Amhara Region’s public health facilities reveals a complex landscape marked by significant achievements, persistent challenges, and opportunities for improvement. This discussion synthesizes key findings to highlight the system’s strengths, weaknesses, and implications for improvement.

The study indicates that most health facilities (96.1%) had operational electric power systems during the survey. However, nearly a third of these facilities experienced power interruptions, underscoring vulnerabilities in infrastructure reliability. This finding aligns with the infrastructure challenges in low-income settings, where power instability disrupts LMIS operations. The presence of backup generators in 61.8% of facilities reflects a response to this challenge but indicates that reliance on backup power needs to be uniformly adequate. The unreliable power supply system can significantly impact the functioning of the LMIS, disrupt data transmission, and necessitate sending reports via transportation methods instead. This, in turn, poses another challenge, as the majority of health facilities lack dedicated transportation methods for sending LMIS reports to higher levels (20).

Similarly, internet connectivity is another critical infrastructure component, available in only 42.2% of the facilities, with limited access, particularly in pharmacy departments. This finding aligns with Endeshaw et al. (24), who identified poor internet infrastructure as a barrier to effective LMIS in health facilities. Additionally, the shortage of computers dedicated to LMIS activities in health facilities directly affects the system’s functionality, which is consistent with the findings of Shewarega et al. (23) and Alemu et al. (22), who reported insufficient LMIS resources and technological gaps in healthcare facilities. This limitation restricts real-time data reporting and hampers the potential benefits of digital systems like HCMIS. Efforts to enhance connectivity infrastructure are essential to leveraging the full capabilities of digital LMIS, ensuring timely and accurate information flow across healthcare levels (5, 17).

Human resources dedicated to supply chain management are limited, with an average of 1.65 professionals per facility. Additionally, only 60.8% of facilities have designated supply chain coordinators, exposing gaps in leadership and oversight crucial for effective health supply chain practices and LMIS utilization. This is consistent with findings from a study in the Oromia Region, which identified a shortage of skilled human resources as a major bottleneck in LMIS performance, and with Fenta (33), which highlighted human resource challenges as key factors affecting supply chain performance in Ethiopia (33, 34).

The study reveals significant variability in the availability and utilization of LMIS forms. Essential forms such as the IFRR and RRF are widely available and used, whereas others, like the HCMIS Guide/Manual, are less accessible. This disparity underscores ongoing challenges in standardizing and integrating LMIS tools across facilities. The high utilization of Bin Cards and Stock Record Cards, despite their lower availability, indicates practical adaptations to resource limitations, aligning with findings from Bekele and Anbessa (26). The low availability and utilization of LMIS forms directly impact LMIS performance and decision-making, ultimately affecting the supply efficiency of health commodities.

The study indicates that 75.5% of facilities submit reports to the Woreda Health Office, with a significant portion reporting to EPSS. However, only 37.3% received written periodic feedback, and 71.6% received supportive supervision within the past year. These figures suggest that while reporting mechanisms are in place, feedback and supervisory support are insufficient, reflecting similar concerns raised in a study conducted in North West Ethiopia about the impact of inadequate feedback and supervision on LMIS performance (29). Strengthening communication channels and enhancing supervisory support are essential for continuous improvements in LMIS (34). Furthermore, the involvement of multiple stakeholders in supervision highlights the critical role of collaboration in sustaining LMIS performance improvements. Most importantly, digitizing the LMIS system and modernizing communication channels for reporting and feedback could significantly support the LMIS overall performance.

The discrepancy between the high utilization of reporting forms and the low percentage of facilities receiving timely feedback indicates a gap in effectively using information to improve practices. This issue mirrors the findings of other studies which highlighted difficulties in leveraging data for decision-making and is consistent with similar concerns noted regarding the underutilization of available data in enhancing health system performance (24, 27). Addressing this gap is crucial, as timely feedback can empower health facilities to improve LMIS performance and enhance overall health outcomes through improving availability of essential health commodities.

## 5 Conclusion and recommendation

The Logistics Management Information System performance evaluation in Amhara Region’s public health facilities highlights a blend of successes and ongoing challenges. While essential forms like IFRR and RRF are widely available and power systems generally operational, critical issues persist, including frequent power interruptions, inadequate internet connectivity, insufficient computer resources, and inconsistent availability and utilization of LMIS forms. The IFRR reporting rate as per the IPLS 2 weeks schedule was found to be 67.04%. Of the facilities using the RRF, 51% placed emergency orders at least once per year. The need for more dedicated personnel for supply chain management and uneven supervisory support further compound these problems. To address these issues, investing in reliable power solutions and improving internet infrastructure is crucial; expanding computer access, standardizing LMIS tools, increasing staffing and training, and enhancing feedback and supervisory mechanisms are crucial. Collaborative efforts among stakeholders are vital to overcoming these barriers and enhancing the effectiveness of the LMIS. Digitizing the LMIS and increasing the utilization of its information for decision-making can significantly improve the health system’s performance, ensuring better access to essential medicines and improved health outcomes.

## Data Availability

The raw data supporting the conclusions of this article will be made available by the authors, without undue reservation.
